# (3-Oxo-3*H*-benzo[*f*]chromen-1-yl)methyl piperidine-1-carbodithio­ate

**DOI:** 10.1107/S1600536812042870

**Published:** 2012-10-20

**Authors:** O. Kotresh, K. Mahesh Kumar, N. M. Mahabaleshwaraiah, H. K. Arunkashi, H. C. Devarajegowda

**Affiliations:** a Department of Chemistry, Karnatak University’s Karnatak Science College, Dharwad, Karnataka 580 001, India; b Department of Physics, Yuvaraja’s College (Constituent College), University of Mysore, Mysore 570 005, Karnataka, India

## Abstract

In the title compound, C_20_H_19_N O_2_S_2_,the 3*H*benzo-chromene ring system is nearly planar, with a maximum deviation of 0.036 (2) Å, and the piperidine ring adopts a chair conformation: the bond-angle sum for its N atom is 358.7°. The dihedral angle between the 3*H-*benzo[*f*]chromene ring and the piperidine ring is 89.07 (8)°. In the crystal, C—H⋯O hydrogen bonds lead to [010] *C*(6) chains and weak aromatic π–π inter­actions between the fused pyran ring and fused benzene ring of benzochromene [centroid–centroid distance = 3.652 (1) Å] are also observed.

## Related literature

For a related structure, background to coumarins and details of the synthesis of the title compound, see: Kumar *et al.* (2012 ▶).
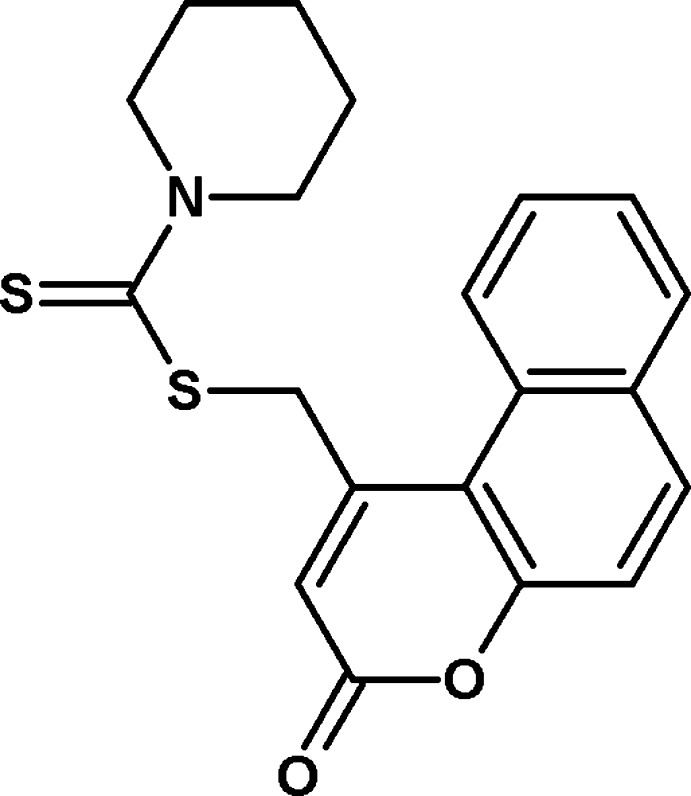


## Experimental

### 

#### Crystal data

C_20_H_19_NO_2_S_2_
*M* *_r_* = 369.48
Monoclinic, 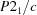

*a* = 12.4508 (3) Å
*b* = 10.1924 (3) Å
*c* = 14.0188 (4) Å
β = 100.953 (2)°
*V* = 1746.63 (8) Å^3^
*Z* = 4
Mo *K*α radiation
μ = 0.32 mm^−1^
*T* = 296 K
0.24 × 0.20 × 0.12 mm

#### Data collection

Bruker SMART CCD diffractometer
Absorption correction: multi-scan (*SADABS*; Sheldrick, 2007 ▶) *T*_min_ = 0.770, *T*_max_ = 1.000
12000 measured reflections
2993 independent reflections
2380 reflections with *I* > 2σ(*I*)
*R*_int_ = 0.029

#### Refinement

*R*[*F* ^2^ > 2σ(*F* ^2^)] = 0.034
*wR*(*F* ^2^) = 0.082
*S* = 1.06
2993 reflections
226 parameters
H-atom parameters constrained
Δρ_max_ = 0.16 e Å^−3^
Δρ_min_ = −0.14 e Å^−3^

### 

Data collection: *SMART* (Bruker, 2001 ▶); cell refinement: *SAINT* (Bruker, 2001 ▶); data reduction: *SAINT*; program(s) used to solve structure: *SHELXS97* (Sheldrick, 2008 ▶); program(s) used to refine structure: *SHELXL97* (Sheldrick, 2008 ▶); molecular graphics: *ORTEP-3* (Farrugia, 1997 ▶); software used to prepare material for publication: *SHELXL97*.

## Supplementary Material

Crystal structure: contains datablock(s) I, global. DOI: 10.1107/S1600536812042870/hb6973sup1.cif

Structure factors: contains datablock(s) I. DOI: 10.1107/S1600536812042870/hb6973Isup2.hkl

Supplementary material file. DOI: 10.1107/S1600536812042870/hb6973Isup3.cml

Additional supplementary materials:  crystallographic information; 3D view; checkCIF report

## Figures and Tables

**Table 1:** Hydrogen-bond geometry (Å, °)

*D*—H⋯*A*	*D*—H	H⋯*A*	*D*⋯*A*	*D*—H⋯*A*
C11—H11⋯O4^i^	0.93	2.56	3.308 (2)	138

Symmetry code: (i) 
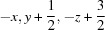
.

**Fig. 1.**
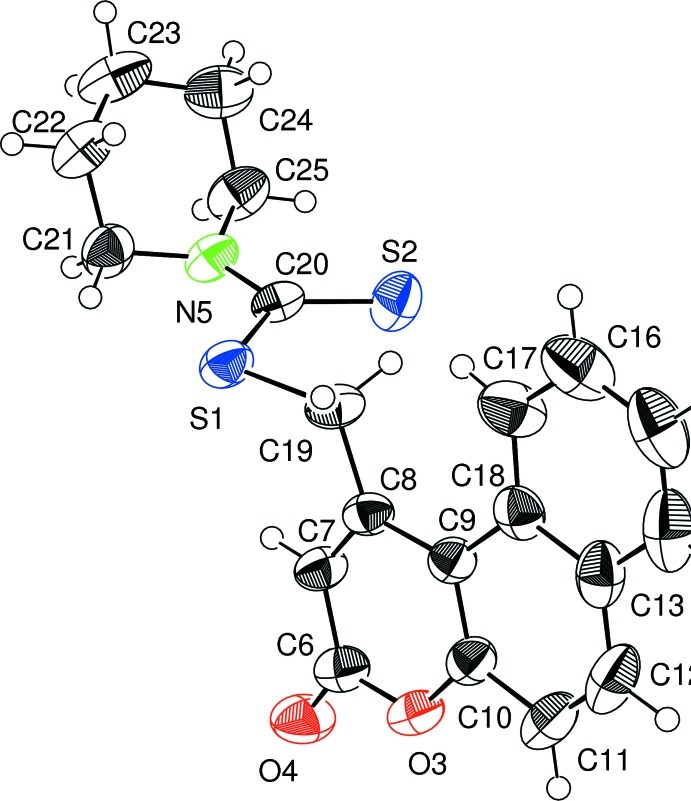
The molecular structure of the title compound. Displacement ellipsoids are drawn at the 50% probability level. Hydrogen atoms are shown as spheres of arbitrary radius.

## supplementary crystallographic information

### Comment

As part of our ongoing studies of coumarins (or 2*H*-chromen-2-ones) with possible biological activities (Kumar *et al.*, 2012), we now describe the structure of the title compound, (I).

The asymmetric unit of (I) is shown in Fig. 1. The 3*H*benzo- chromene ring system (O3/C6–C18) is essentially planar, with a maximum deviation of 0.036 (2) Å for atom C15 and the piperidine ring adopts a chair conformation. The dihedral angle between the 3*H*benzo- chromene (O3/C6–C18) ring and the piperidine (N5/C21–C25) ring is 81.07 (8)°.

In the crystal structure, (Fig. 2), C11—H11···O4 hydrogen bonds and π–π interactions between the fused pyran ring (O3/C6–C10) and fused benzene(C13–C18) ring of benzo-chromene [shortest centroid–centroid distance = 3.652 (1) Å] are observed.

### Experimental

This compound was prepared according to the reported method (Kumar *et al.*, 2012). Colourless needles of the title compound were grown from a mixed solution of EtOH / CHCl_3_(V/V = 1/1) by slow evaporation at room temperature. Yield= 85%, m.p. 455 K.

### Refinement

All H atoms were positioned geometrically, with C—H = 0.93 Å for aromatic H and C—H = 0.97 Å for methylene H and refined using a riding model with *U*_iso_(H) = 1.2*U*_eq_(C) foraromatic and methylene H.

### Crystal data

**Table:**

C_20_H_19_NO_2_S_2_	*F*(000) = 776
*M**_r_* = 369.48	*D*_x_ = 1.405 Mg m^−^^3^
Monoclinic, *P*2_1_/*c*	Melting point: 455 K
Hall symbol: -P 2ybc	Mo *K*α radiation, λ = 0.71073 Å
*a* = 12.4508 (3) Å	Cell parameters from 2993 reflections
*b* = 10.1924 (3) Å	θ = 1.7–24.9°
*c* = 14.0188 (4) Å	µ = 0.32 mm^−^^1^
β = 100.953 (2)°	*T* = 296 K
*V* = 1746.63 (8) Å^3^	Plate, colourless
*Z* = 4	0.24 × 0.20 × 0.12 mm

### Data collection

**Table:**

Bruker SMART CCD diffractometer	2993 independent reflections
Radiation source: fine-focus sealed tube	2380 reflections with *I* > 2σ(*I*)
Graphite monochromator	*R*_int_ = 0.029
ω and φ scans	θ_max_ = 24.9°, θ_min_ = 1.7°
Absorption correction: multi-scan (*SADABS*; Sheldrick, 2007)	*h* = −14→14
*T*_min_ = 0.770, *T*_max_ = 1.000	*k* = −12→10
12000 measured reflections	*l* = −16→16

### Refinement

**Table:**

Refinement on *F*^2^	Primary atom site location: structure-invariant direct methods
Least-squares matrix: full	Secondary atom site location: difference Fourier map
*R*[*F*^2^ > 2σ(*F*^2^)] = 0.034	Hydrogen site location: inferred from neighbouring sites
*wR*(*F*^2^) = 0.082	H-atom parameters constrained
*S* = 1.06	*w* = 1/[σ^2^(*F*_o_^2^) + (0.0374*P*)^2^ + 0.1604*P*] where *P* = (*F*_o_^2^ + 2*F*_c_^2^)/3
2993 reflections	(Δ/σ)_max_ < 0.001
226 parameters	Δρ_max_ = 0.16 e Å^−^^3^
0 restraints	Δρ_min_ = −0.14 e Å^−^^3^

### Special details

**Table:**

Experimental. IR (KBr): 655 cm^-1^(C—S), 1235 cm^-1^(C=S), 1007 cm^-1^(C—O), 877 cm^-1^(C—N),1137 cm^-1^(C—O—C), 1720 cm^-1^(C=O). GCMS: m/e: 369. 1H NMR (400 MHz, CDCl_3_, \?, p.p.m.): 1.74(s, 6H, Piperidine-CH_2_), 3.90(s, 2H, Piperidine-CH_2_), 4.32(s, 2H Piperidine-CH_2_), 4.85(s, 2H, Methylene-CH_2_), 6.67(s,1*H*, Ar—H), 7.64(s, 2H, Ar—H), 7.65(s, 2H, Ar—H), 7.88(m,1*H*, Ar—H), 8.56(m,1*H*, Ar—H). Elemental analysis for C_20_H_19_NO_2_S_2_: C, 64.93; H, 5.11; N, 3.68.
Geometry. All s.u.'s (except the s.u. in the dihedral angle between two l.s. planes) are estimated using the full covariance matrix. The cell s.u.'s are taken into account individually in the estimation of s.u.'s in distances, angles and torsion angles; correlations between s.u.'s in cell parameters are only used when they are defined by crystal symmetry. An approximate (isotropic) treatment of cell s.u.'s is used for estimating s.u.'s involving l.s. planes.
Refinement. Refinement of *F*^2^ against ALL reflections. The weighted *R*-factor *wR* and goodness of fit *S* are based on *F*^2^, conventional *R*-factors *R* are based on *F*, with *F* set to zero for negative *F*^2^. The threshold expression of *F*^2^ > 2σ(*F*^2^) is used only for calculating *R*-factors(gt) *etc*. and is not relevant to the choice of reflections for refinement. *R*-factors based on *F*^2^ are statistically about twice as large as those based on *F*, and *R*- factors based on ALL data will be even larger.

### Fractional atomic coordinates and isotropic or equivalent isotropic displacement parameters (Å^2^)

**Table:**

	*x*	*y*	*z*	*U*_iso_*/*U*_eq_	
S1	0.27800 (4)	0.69065 (5)	0.37375 (3)	0.05055 (15)	
S2	0.48792 (4)	0.78586 (6)	0.49758 (3)	0.06250 (17)	
O3	0.09397 (10)	0.81457 (13)	0.67448 (8)	0.0520 (3)	
O4	0.12823 (11)	0.60307 (14)	0.68426 (9)	0.0669 (4)	
N5	0.46698 (11)	0.57768 (15)	0.38149 (9)	0.0502 (4)	
C6	0.13705 (14)	0.7038 (2)	0.64137 (12)	0.0502 (5)	
C7	0.18621 (14)	0.72190 (18)	0.55883 (12)	0.0469 (4)	
H7	0.2160	0.6487	0.5340	0.056*	
C8	0.19268 (12)	0.83761 (17)	0.51406 (11)	0.0412 (4)	
C9	0.15023 (12)	0.95573 (17)	0.55367 (11)	0.0410 (4)	
C10	0.10299 (13)	0.93577 (18)	0.63419 (11)	0.0448 (4)	
C11	0.05735 (16)	1.0358 (2)	0.68248 (13)	0.0619 (6)	
H11	0.0263	1.0168	0.7362	0.074*	
C12	0.05917 (17)	1.1592 (2)	0.64997 (15)	0.0691 (6)	
H12	0.0297	1.2258	0.6824	0.083*	
C13	0.10462 (15)	1.19123 (19)	0.56782 (14)	0.0574 (5)	
C14	0.10225 (19)	1.3216 (2)	0.53383 (19)	0.0753 (7)	
H14	0.0715	1.3865	0.5668	0.090*	
C15	0.14331 (19)	1.3548 (2)	0.4550 (2)	0.0779 (7)	
H15	0.1404	1.4412	0.4333	0.093*	
C16	0.18980 (18)	1.2583 (2)	0.40712 (19)	0.0778 (7)	
H16	0.2185	1.2806	0.3527	0.093*	
C17	0.19479 (16)	1.1313 (2)	0.43759 (16)	0.0662 (6)	
H17	0.2279	1.0695	0.4039	0.079*	
C18	0.15154 (12)	1.09039 (18)	0.51844 (12)	0.0473 (4)	
C19	0.24178 (14)	0.84276 (18)	0.42314 (12)	0.0491 (4)	
H19A	0.3070	0.8968	0.4371	0.059*	
H19B	0.1901	0.8873	0.3731	0.059*	
C20	0.42141 (14)	0.67877 (17)	0.41861 (11)	0.0438 (4)	
C21	0.41030 (16)	0.48928 (19)	0.30567 (13)	0.0568 (5)	
H21A	0.3318	0.5002	0.2989	0.068*	
H21B	0.4282	0.3990	0.3239	0.068*	
C22	0.44414 (17)	0.5192 (2)	0.21075 (13)	0.0663 (6)	
H22A	0.4193	0.6065	0.1894	0.080*	
H22B	0.4098	0.4571	0.1619	0.080*	
C23	0.56693 (18)	0.5117 (2)	0.22032 (15)	0.0780 (7)	
H23A	0.5871	0.5421	0.1606	0.094*	
H23B	0.5903	0.4211	0.2303	0.094*	
C24	0.62477 (16)	0.5941 (2)	0.30422 (15)	0.0700 (6)	
H24A	0.7030	0.5792	0.3131	0.084*	
H24B	0.6112	0.6862	0.2892	0.084*	
C25	0.58614 (14)	0.5613 (2)	0.39697 (13)	0.0630 (6)	
H25A	0.6055	0.4715	0.4158	0.076*	
H25B	0.6212	0.6189	0.4488	0.076*	

### Atomic displacement parameters (Å^2^)

**Table:**

	*U*^11^	*U*^22^	*U*^33^	*U*^12^	*U*^13^	*U*^23^
S1	0.0437 (3)	0.0616 (3)	0.0487 (3)	0.0059 (2)	0.01482 (19)	−0.0062 (2)
S2	0.0606 (3)	0.0702 (4)	0.0536 (3)	0.0040 (3)	0.0032 (2)	−0.0135 (2)
O3	0.0525 (7)	0.0653 (9)	0.0419 (6)	0.0039 (6)	0.0182 (5)	0.0000 (6)
O4	0.0801 (9)	0.0660 (10)	0.0613 (8)	0.0012 (8)	0.0303 (7)	0.0170 (8)
N5	0.0491 (8)	0.0593 (10)	0.0437 (7)	0.0116 (7)	0.0126 (6)	−0.0042 (7)
C6	0.0484 (10)	0.0607 (14)	0.0433 (9)	0.0027 (9)	0.0130 (8)	0.0041 (9)
C7	0.0466 (10)	0.0502 (12)	0.0478 (9)	0.0071 (8)	0.0192 (8)	0.0002 (8)
C8	0.0324 (8)	0.0510 (12)	0.0416 (8)	0.0034 (8)	0.0108 (7)	0.0005 (8)
C9	0.0302 (8)	0.0487 (11)	0.0440 (9)	0.0018 (7)	0.0069 (7)	−0.0048 (8)
C10	0.0384 (9)	0.0544 (12)	0.0411 (9)	0.0026 (8)	0.0062 (7)	−0.0051 (8)
C11	0.0632 (13)	0.0766 (17)	0.0473 (10)	0.0106 (11)	0.0143 (9)	−0.0165 (10)
C12	0.0717 (14)	0.0691 (17)	0.0649 (13)	0.0160 (12)	0.0090 (11)	−0.0274 (12)
C13	0.0477 (11)	0.0527 (13)	0.0657 (12)	0.0018 (9)	−0.0043 (9)	−0.0156 (10)
C14	0.0724 (15)	0.0483 (14)	0.0958 (17)	0.0052 (11)	−0.0076 (13)	−0.0186 (12)
C15	0.0727 (15)	0.0456 (14)	0.1077 (18)	−0.0077 (11)	−0.0022 (13)	0.0075 (13)
C16	0.0667 (14)	0.0626 (16)	0.1065 (17)	−0.0027 (12)	0.0223 (13)	0.0195 (13)
C17	0.0600 (12)	0.0517 (14)	0.0930 (15)	0.0079 (10)	0.0298 (11)	0.0149 (11)
C18	0.0316 (9)	0.0487 (12)	0.0594 (10)	−0.0001 (8)	0.0033 (7)	−0.0035 (9)
C19	0.0467 (10)	0.0546 (12)	0.0507 (9)	0.0123 (8)	0.0209 (8)	0.0073 (8)
C20	0.0484 (10)	0.0524 (12)	0.0334 (8)	0.0075 (8)	0.0151 (7)	0.0053 (7)
C21	0.0619 (12)	0.0504 (12)	0.0602 (11)	0.0073 (9)	0.0172 (9)	−0.0064 (9)
C22	0.0743 (14)	0.0786 (16)	0.0473 (10)	0.0168 (11)	0.0150 (9)	−0.0072 (10)
C23	0.0788 (15)	0.106 (2)	0.0560 (12)	0.0296 (13)	0.0302 (11)	0.0052 (12)
C24	0.0554 (12)	0.0809 (17)	0.0787 (14)	0.0189 (11)	0.0252 (10)	0.0102 (12)
C25	0.0504 (11)	0.0821 (16)	0.0569 (11)	0.0239 (10)	0.0112 (9)	−0.0007 (10)

### Geometric parameters (Å, º)

**Table:**

S1—C20	1.7809 (17)	C14—H14	0.9300
S1—C19	1.7904 (18)	C15—C16	1.379 (3)
S2—C20	1.6597 (18)	C15—H15	0.9300
O3—C6	1.368 (2)	C16—C17	1.361 (3)
O3—C10	1.372 (2)	C16—H16	0.9300
O4—C6	1.205 (2)	C17—C18	1.407 (3)
N5—C20	1.329 (2)	C17—H17	0.9300
N5—C25	1.468 (2)	C19—H19A	0.9700
N5—C21	1.468 (2)	C19—H19B	0.9700
C6—C7	1.420 (2)	C21—C22	1.501 (3)
C7—C8	1.346 (2)	C21—H21A	0.9700
C7—H7	0.9300	C21—H21B	0.9700
C8—C9	1.466 (2)	C22—C23	1.511 (3)
C8—C19	1.516 (2)	C22—H22A	0.9700
C9—C10	1.383 (2)	C22—H22B	0.9700
C9—C18	1.460 (2)	C23—C24	1.512 (3)
C10—C11	1.403 (2)	C23—H23A	0.9700
C11—C12	1.339 (3)	C23—H23B	0.9700
C11—H11	0.9300	C24—C25	1.507 (3)
C12—C13	1.414 (3)	C24—H24A	0.9700
C12—H12	0.9300	C24—H24B	0.9700
C13—C14	1.410 (3)	C25—H25A	0.9700
C13—C18	1.425 (2)	C25—H25B	0.9700
C14—C15	1.346 (3)		
			
C20—S1—C19	103.40 (8)	C17—C18—C9	125.56 (17)
C6—O3—C10	122.36 (13)	C13—C18—C9	118.80 (16)
C20—N5—C25	121.67 (16)	C8—C19—S1	117.83 (12)
C20—N5—C21	125.25 (15)	C8—C19—H19A	107.8
C25—N5—C21	111.76 (14)	S1—C19—H19A	107.8
O4—C6—O3	117.06 (16)	C8—C19—H19B	107.8
O4—C6—C7	127.68 (18)	S1—C19—H19B	107.8
O3—C6—C7	115.26 (16)	H19A—C19—H19B	107.2
C8—C7—C6	124.55 (16)	N5—C20—S2	125.10 (13)
C8—C7—H7	117.7	N5—C20—S1	112.86 (13)
C6—C7—H7	117.7	S2—C20—S1	122.04 (10)
C7—C8—C9	119.04 (14)	N5—C21—C22	109.86 (16)
C7—C8—C19	119.48 (15)	N5—C21—H21A	109.7
C9—C8—C19	121.48 (14)	C22—C21—H21A	109.7
C10—C9—C18	116.62 (15)	N5—C21—H21B	109.7
C10—C9—C8	115.34 (15)	C22—C21—H21B	109.7
C18—C9—C8	128.03 (14)	H21A—C21—H21B	108.2
O3—C10—C9	123.31 (15)	C21—C22—C23	111.14 (16)
O3—C10—C11	112.56 (15)	C21—C22—H22A	109.4
C9—C10—C11	124.12 (18)	C23—C22—H22A	109.4
C12—C11—C10	118.93 (19)	C21—C22—H22B	109.4
C12—C11—H11	120.5	C23—C22—H22B	109.4
C10—C11—H11	120.5	H22A—C22—H22B	108.0
C11—C12—C13	121.94 (18)	C22—C23—C24	111.43 (16)
C11—C12—H12	119.0	C22—C23—H23A	109.3
C13—C12—H12	119.0	C24—C23—H23A	109.3
C14—C13—C12	120.4 (2)	C22—C23—H23B	109.3
C14—C13—C18	120.0 (2)	C24—C23—H23B	109.3
C12—C13—C18	119.57 (18)	H23A—C23—H23B	108.0
C15—C14—C13	121.8 (2)	C25—C24—C23	111.42 (19)
C15—C14—H14	119.1	C25—C24—H24A	109.3
C13—C14—H14	119.1	C23—C24—H24A	109.3
C14—C15—C16	118.7 (2)	C25—C24—H24B	109.3
C14—C15—H15	120.6	C23—C24—H24B	109.3
C16—C15—H15	120.6	H24A—C24—H24B	108.0
C17—C16—C15	121.6 (2)	N5—C25—C24	109.06 (15)
C17—C16—H16	119.2	N5—C25—H25A	109.9
C15—C16—H16	119.2	C24—C25—H25A	109.9
C16—C17—C18	122.2 (2)	N5—C25—H25B	109.9
C16—C17—H17	118.9	C24—C25—H25B	109.9
C18—C17—H17	118.9	H25A—C25—H25B	108.3
C17—C18—C13	115.63 (18)		

### Hydrogen-bond geometry (Å, º)

**Table:**

*D*—H···*A*	*D*—H	H···*A*	*D*···*A*	*D*—H···*A*
C11—H11···O4^i^	0.93	2.56	3.308 (2)	138

Symmetry code: (i) −*x*, *y*+1/2, −*z*+3/2.
